# μ-Actetato-1:2κ^2^ *O*:*O*′-tribromido-2κ^3^ *Br*-(5,5,7,12,12,14-hexa­methyl-1,4,8,11-tetra­aza­cyclo­tetra­deca-1,7-diene-1κ^4^ *N*)dizinc(II)

**DOI:** 10.1107/S1600536810033702

**Published:** 2010-09-30

**Authors:** HuaFeng Li, FeiFei Shi, Rong Rong

**Affiliations:** a Ordered Matter Science Research Center, College of Chemistry and Chemical Engineering, Southeast University, Nanjing 210096, People’s Republic of China; b Department of Chemistry, Key Laboratory of Medicinal Chemistry for Natural Resource, Ministry of Education, Yunnan University, Kunming 650091, People’s Republic of China

## Abstract

In the title compound, [Zn_2_Br_3_(CH_3_COO)(C_16_H_32_N_4_)], one Zn^II^ atom has a distorted square-planar coordination formed by the four macrocyclic N atoms with an acetate O atom in the apical position and the other Zn^II^ atom has a tetra­hedral coordination environment formed by three Br atoms and one O acetate atom. The two Zn^II^ atoms are linked by an acetate bridge. In the crystal, mol­ecules are linked into centrosymmetric dimers with graph-set motifs *R*_2_^2^(16) by an N—H⋯Br inter­action. The mol­ecular configuration is stabilized by an intra­molecular N—H⋯Br hydrogen bond.

## Related literature

For related macrocyclic complexes, see: Whimp *et al.* (1970 ▶); Yang (2005 ▶); Tebbe *et al.* (1985 ▶). The unsubstituted parent compound exists in the zwitterionic form, see: Spirlet *et al.* (1991 ▶); Maurya *et al.* (1991 ▶). For the preparation of the precursor complex C_16_H_32_N_4_·2HBr·2H_2_O, see: Hay *et al.* (1975 ▶). For hydrogen-bond motifs, see: Bernstein *et al.* (1995 ▶).
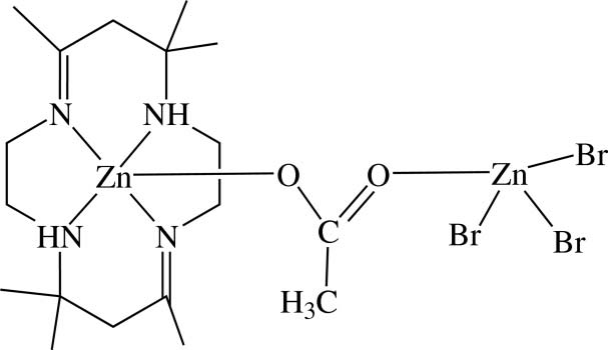


## Experimental

### 

#### Crystal data

[Zn_2_Br_3_(C_2_H_3_O_2_)(C_16_H_32_N_4_)]
*M* *_r_* = 709.97
Monoclinic, 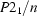

*a* = 10.2964 (8) Å
*b* = 13.6985 (13) Å
*c* = 18.5235 (18) Å
β = 92.280 (1)°
*V* = 2610.6 (4) Å^3^
*Z* = 4
Mo *K*α radiation
μ = 6.45 mm^−1^
*T* = 298 K
0.43 × 0.42 × 0.22 mm

#### Data collection

Rigaku SCXmini diffractometer
Absorption correction: multi-scan (*CrystalClear*; Rigaku, 2005 ▶) *T*_min_ = 0.240, *T*_max_ = 0.428
13032 measured reflections
4608 independent reflections
2923 reflections with *I* > 2σ(*I*)
*R*_int_ = 0.038

#### Refinement

*R*[*F* ^2^ > 2σ(*F* ^2^)] = 0.032
*wR*(*F* ^2^) = 0.067
*S* = 0.88
4608 reflections
269 parameters
H-atom parameters constrained
Δρ_max_ = 0.57 e Å^−3^
Δρ_min_ = −0.55 e Å^−3^

### 

Data collection: *CrystalClear* (Rigaku, 2005 ▶); cell refinement: *CrystalClear*; data reduction: *CrystalClear*; program(s) used to solve structure: *SHELXS97* (Sheldrick, 2008 ▶); program(s) used to refine structure: *SHELXL97* (Sheldrick, 2008 ▶); molecular graphics: *SHELXTL* (Sheldrick, 2008 ▶); software used to prepare material for publication: *SHELXTL*.

## Supplementary Material

Crystal structure: contains datablocks I, global. DOI: 10.1107/S1600536810033702/bx2283sup1.cif

Structure factors: contains datablocks I. DOI: 10.1107/S1600536810033702/bx2283Isup2.hkl

Additional supplementary materials:  crystallographic information; 3D view; checkCIF report

## Figures and Tables

**Table 1:** Hydrogen-bond geometry (Å, °)

*D*—H⋯*A*	*D*—H	H⋯*A*	*D*⋯*A*	*D*—H⋯*A*
N3—H3⋯Br2^i^	0.91	2.80	3.549 (3)	140
N1—H1⋯Br1	0.91	2.74	3.641 (3)	171

Symmetry code: (i) 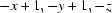.

**Fig. 1.**
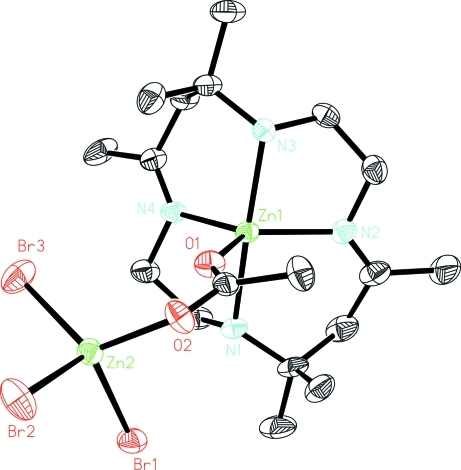
The asymmetric structure of the title molecule, with the atom-numbering scheme. Displacement ellipsoids are drawn at the 30% probability level.

## supplementary crystallographic information

### Comment

The structures of several related macrocyclic complexes have been reported (Whimp *et al.*, 1970; Yang, 2005; Tebbe *et al.*, 1985). The unsubstituted parent compound exists in the zwitterionic form (Maurya *et al.*, 1991; Spirlet *et al.*, 1991). The zinc teraazamacrocyclic complex cation, [Zn(C_16_H_32_N_4_)] ^2+^, can combine with different anions to form many kinds of structures.

We herein report the crystal structure of a new compound synthesized by reaction of Zn(CH_3_COO)_2_.2H_2_O and the complex C_18_H_32_N_4_.2HBr.2H_2_O, Fig.1.  The structural analysis reveals that the title complex is formed by a discrete neutral dinuclear C_18_H_35_N_4_O_2_Br_3_Zn_2_ molecule consisting of two Zn atoms bridged by an acetate with the distance of 6.512 (1) Å between the them. Zn(1) is five-coordinated by the four macrocyclic N atoms with acetate O atom as an apical ligand while that the other Zn atom is in a tetrahedron coordinate environment formed by three bromine atoms and one O acetate atom. The average Zn—*N*(amine) bond distance of 2.1546 (5)Å and Zn—*N*(imine) bond distance of 2.0582 (5) Å). The average Zn—Br bond distance of 2.4070 (6) Å, the Zn(1)—O(1) bond distance of` 2.0030 (1) Å and the Zn(2)—O(2) bond distance of` 1.9967 (1) Å). In the crystal the molecules are linked into centrosymmetric dimers with graph-set notation *R^2^_2_*(16) motifs by a N—H···Br interaction, centred at [1/2,1/2,0] (Bernstein *et al.*, 1995), Fig. 2. The molecular conformation is stabilized by one intramolecular N—H···Br hydrogen bond. Table 1.

### Experimental

All chemicals were of reagent grade and were used as received without further purification. The precursor complex C_18_H_32_N_4_.2HBr.2H_2_O was prepared by the literature method (Hay *et al.*,1975). To a 10 ml me thanol solution of Zn(CH_3_COO)_2_.2H_2_O(0.2 mmol,0.039 g), a 5 ml methanol solution of C_18_H_32_N_4_.2HBr.2H_2_O (0.2 mmol,0.0957 g) was added dropwise with stirring. The resulting solution was continuously stirred for about 30 min. Colourless crystals suitable for X-ray analysis were obtained by slow evaporation at room temperature over several days.

### Refinement

All H atoms were refined as riding on their parent atoms, with distances of 0.91 (NH),  0.97 (CH_2_) and 0.96 (CH_3_) Å from the parent C and N atoms, with U_iso_(H) = 1.2U_eq_(CH_2_, N) or 1.5U_eq_(CH_3_).

### Crystal data

**Table:**

[Zn_2_Br_3_(C_2_H_3_O_2_)(C_16_H_32_N_4_)]	*F*(000) = 1408
*M**_r_* = 709.97	*D*_x_ = 1.806 Mg m^−^^3^
Monoclinic, *P*2_1_/*n*	Mo *K*α radiation, λ = 0.71073 Å
Hall symbol: -P 2yn	Cell parameters from 3476 reflections
*a* = 10.2964 (8) Å	θ = 2.3–27.5°
*b* = 13.6985 (13) Å	µ = 6.45 mm^−^^1^
*c* = 18.5235 (18) Å	*T* = 298 K
β = 92.280 (1)°	Prism, purple
*V* = 2610.6 (4) Å^3^	0.43 × 0.42 × 0.22 mm
*Z* = 4	

### Data collection

**Table:**

Rigaku SCXmini diffractometer	4608 independent reflections
Radiation source: fine-focus sealed tube	2923 reflections with *I* > 2σ(*I*)
graphite	*R*_int_ = 0.038
Detector resolution: 13.6612 pixels mm^-1^	θ_max_ = 25.0°, θ_min_ = 1.9°
thin–slice ω scans	*h* = −12→9
Absorption correction: multi-scan (*CrystalClear*; Rigaku, 2005)	*k* = −16→14
*T*_min_ = 0.240, *T*_max_ = 0.428	*l* = −22→17
13032 measured reflections	

### Refinement

**Table:**

Refinement on *F*^2^	Primary atom site location: structure-invariant direct methods
Least-squares matrix: full	Secondary atom site location: difference Fourier map
*R*[*F*^2^ > 2σ(*F*^2^)] = 0.032	Hydrogen site location: inferred from neighbouring sites
*wR*(*F*^2^) = 0.067	H-atom parameters constrained
*S* = 0.88	*w* = 1/[σ^2^(*F*_o_^2^) + (0.0294*P*)^2^] where *P* = (*F*_o_^2^ + 2*F*_c_^2^)/3
4608 reflections	(Δ/σ)_max_ = 0.001
269 parameters	Δρ_max_ = 0.57 e Å^−^^3^
0 restraints	Δρ_min_ = −0.55 e Å^−^^3^

### Special details

**Table:**

Geometry. All e.s.d.'s (except the e.s.d. in the dihedral angle between two l.s. planes) are estimated using the full covariance matrix. The cell e.s.d.'s are taken into account individually in the estimation of e.s.d.'s in distances, angles and torsion angles; correlations between e.s.d.'s in cell parameters are only used when they are defined by crystal symmetry. An approximate (isotropic) treatment of cell e.s.d.'s is used for estimating e.s.d.'s involving l.s. planes.
Refinement. Refinement of *F*^2^ against ALL reflections. The weighted *R*-factor *wR* and goodness of fit *S* are based on *F*^2^, conventional *R*-factors *R* are based on *F*, with *F* set to zero for negative *F*^2^. The threshold expression of *F*^2^ > σ(*F*^2^) is used only for calculating *R*-factors(gt) *etc*. and is not relevant to the choice of reflections for refinement. *R*-factors based on *F*^2^ are statistically about twice as large as those based on *F*, and *R*- factors based on ALL data will be even larger.

### Fractional atomic coordinates and isotropic or equivalent isotropic displacement parameters (Å^2^)

**Table:**

	*x*	*y*	*z*	*U*_iso_*/*U*_eq_	
Zn1	0.41999 (5)	0.72812 (3)	0.14536 (3)	0.03733 (14)	
Zn2	0.62870 (5)	0.70274 (3)	−0.07545 (3)	0.04247 (15)	
Br1	0.76873 (5)	0.82052 (4)	−0.01332 (3)	0.05506 (16)	
Br2	0.72235 (6)	0.62875 (4)	−0.18003 (3)	0.06807 (19)	
Br3	0.43184 (6)	0.78165 (4)	−0.11309 (3)	0.07402 (19)	
N1	0.5700 (3)	0.8386 (2)	0.14436 (19)	0.0423 (9)	
H1	0.6128	0.8291	0.1029	0.051*	
N2	0.4572 (4)	0.6965 (3)	0.25310 (19)	0.0469 (10)	
N3	0.2615 (3)	0.6280 (2)	0.15567 (17)	0.0368 (9)	
H3	0.2904	0.5687	0.1411	0.044*	
N4	0.3048 (4)	0.8384 (2)	0.10354 (19)	0.0432 (10)	
O1	0.5078 (3)	0.6609 (2)	0.06459 (16)	0.0492 (8)	
O2	0.6337 (3)	0.58620 (19)	−0.01065 (15)	0.0505 (9)	
C1	0.6712 (5)	0.8346 (4)	0.2045 (3)	0.0527 (13)	
C2	0.6081 (5)	0.8285 (4)	0.2784 (2)	0.0676 (16)	
H2A	0.6758	0.8394	0.3154	0.081*	
H2B	0.5472	0.8823	0.2811	0.081*	
C3	0.5369 (5)	0.7362 (4)	0.2978 (3)	0.0594 (14)	
C4	0.5646 (5)	0.6981 (5)	0.3728 (3)	0.096 (2)	
H4A	0.4887	0.7057	0.4008	0.144*	
H4B	0.6355	0.7341	0.3951	0.144*	
H4C	0.5873	0.6303	0.3705	0.144*	
C5	0.7534 (5)	0.7430 (4)	0.1919 (3)	0.0635 (15)	
H5A	0.6994	0.6861	0.1941	0.095*	
H5B	0.8218	0.7389	0.2285	0.095*	
H5C	0.7904	0.7468	0.1452	0.095*	
C6	0.7621 (5)	0.9236 (4)	0.2029 (3)	0.0804 (18)	
H6A	0.7923	0.9320	0.1550	0.121*	
H6B	0.8351	0.9134	0.2360	0.121*	
H6C	0.7157	0.9809	0.2168	0.121*	
C7	0.3741 (5)	0.6141 (3)	0.2740 (2)	0.0548 (14)	
H7A	0.3624	0.6157	0.3257	0.066*	
H7B	0.4157	0.5528	0.2624	0.066*	
C8	0.2441 (5)	0.6206 (3)	0.2345 (2)	0.0477 (12)	
H8A	0.1930	0.5631	0.2448	0.057*	
H8B	0.1976	0.6775	0.2510	0.057*	
C9	0.1416 (4)	0.6503 (3)	0.1099 (3)	0.0465 (12)	
C10	0.0989 (4)	0.7575 (3)	0.1209 (3)	0.0515 (13)	
H10A	0.0130	0.7649	0.0981	0.062*	
H10B	0.0899	0.7674	0.1723	0.062*	
C11	0.1824 (5)	0.8391 (3)	0.0939 (2)	0.0453 (12)	
C12	0.1073 (5)	0.9219 (3)	0.0586 (3)	0.0803 (19)	
H12A	0.1603	0.9795	0.0590	0.121*	
H12B	0.0302	0.9342	0.0847	0.121*	
H12C	0.0834	0.9047	0.0096	0.121*	
C13	0.1742 (5)	0.6312 (3)	0.0316 (2)	0.0619 (15)	
H13A	0.2465	0.6711	0.0191	0.093*	
H13B	0.1003	0.6467	0.0005	0.093*	
H13C	0.1963	0.5636	0.0259	0.093*	
C14	0.0265 (5)	0.5847 (3)	0.1298 (3)	0.0669 (15)	
H14A	0.0518	0.5174	0.1268	0.100*	
H14B	−0.0460	0.5967	0.0969	0.100*	
H14C	0.0023	0.5991	0.1782	0.100*	
C15	0.3888 (5)	0.9187 (3)	0.0800 (3)	0.0567 (14)	
H15A	0.3389	0.9786	0.0756	0.068*	
H15B	0.4227	0.9036	0.0332	0.068*	
C16	0.4994 (5)	0.9317 (3)	0.1349 (3)	0.0626 (15)	
H16A	0.5581	0.9818	0.1188	0.075*	
H16B	0.4657	0.9522	0.1807	0.075*	
C17	0.5813 (5)	0.5924 (3)	0.0502 (2)	0.0380 (11)	
C18	0.6139 (5)	0.5127 (3)	0.1038 (2)	0.0585 (14)	
H18A	0.5482	0.5100	0.1391	0.088*	
H18B	0.6966	0.5262	0.1274	0.088*	
H18C	0.6177	0.4513	0.0790	0.088*	

### Atomic displacement parameters (Å^2^)

**Table:**

	*U*^11^	*U*^22^	*U*^33^	*U*^12^	*U*^13^	*U*^23^
Zn1	0.0313 (3)	0.0354 (3)	0.0452 (3)	0.0023 (2)	0.0018 (2)	−0.0006 (2)
Zn2	0.0463 (4)	0.0369 (3)	0.0442 (3)	−0.0020 (3)	0.0017 (3)	0.0035 (2)
Br1	0.0495 (4)	0.0584 (3)	0.0572 (3)	−0.0164 (3)	0.0010 (2)	−0.0022 (3)
Br2	0.1039 (5)	0.0423 (3)	0.0601 (3)	0.0021 (3)	0.0300 (3)	0.0018 (3)
Br3	0.0596 (4)	0.0683 (4)	0.0919 (4)	0.0104 (3)	−0.0254 (3)	0.0014 (3)
N1	0.032 (2)	0.045 (2)	0.050 (2)	−0.0014 (18)	0.0042 (19)	−0.0088 (18)
N2	0.043 (3)	0.055 (3)	0.042 (2)	0.000 (2)	0.001 (2)	−0.006 (2)
N3	0.038 (2)	0.0321 (19)	0.040 (2)	0.0040 (17)	0.0038 (18)	−0.0003 (17)
N4	0.033 (3)	0.036 (2)	0.060 (3)	0.0012 (18)	0.004 (2)	0.0044 (18)
O1	0.043 (2)	0.0449 (19)	0.060 (2)	0.0045 (16)	0.0063 (16)	−0.0063 (16)
O2	0.067 (3)	0.0379 (18)	0.048 (2)	0.0049 (16)	0.0190 (18)	0.0034 (15)
C1	0.032 (3)	0.067 (3)	0.058 (3)	−0.012 (3)	0.001 (3)	−0.020 (3)
C2	0.054 (4)	0.093 (4)	0.056 (3)	−0.008 (3)	−0.001 (3)	−0.029 (3)
C3	0.039 (3)	0.092 (4)	0.048 (3)	0.003 (3)	0.009 (3)	−0.022 (3)
C4	0.069 (5)	0.181 (7)	0.038 (3)	−0.027 (4)	−0.007 (3)	0.004 (4)
C5	0.040 (3)	0.094 (4)	0.056 (3)	0.012 (3)	−0.005 (3)	−0.007 (3)
C6	0.052 (4)	0.099 (4)	0.089 (4)	−0.032 (3)	0.000 (3)	−0.024 (4)
C7	0.067 (4)	0.055 (3)	0.043 (3)	−0.003 (3)	0.002 (3)	0.000 (2)
C8	0.051 (4)	0.045 (3)	0.048 (3)	−0.003 (2)	0.016 (3)	−0.003 (2)
C9	0.033 (3)	0.036 (3)	0.071 (4)	−0.002 (2)	0.000 (3)	0.003 (2)
C10	0.032 (3)	0.044 (3)	0.079 (4)	0.009 (2)	0.002 (3)	0.006 (2)
C11	0.040 (3)	0.036 (3)	0.060 (3)	0.001 (2)	−0.001 (3)	−0.004 (2)
C12	0.056 (4)	0.043 (3)	0.139 (5)	0.003 (3)	−0.042 (4)	0.017 (3)
C13	0.069 (4)	0.055 (3)	0.061 (4)	0.002 (3)	−0.016 (3)	−0.004 (3)
C14	0.042 (4)	0.052 (3)	0.107 (4)	−0.010 (3)	−0.001 (3)	0.008 (3)
C15	0.045 (4)	0.037 (3)	0.088 (4)	0.000 (2)	0.002 (3)	0.016 (3)
C16	0.049 (4)	0.039 (3)	0.100 (4)	−0.006 (3)	0.009 (3)	−0.004 (3)
C17	0.042 (3)	0.033 (3)	0.039 (3)	−0.011 (2)	0.000 (2)	−0.001 (2)
C18	0.079 (4)	0.046 (3)	0.051 (3)	0.000 (3)	0.004 (3)	0.008 (2)

### Geometric parameters (Å, °)

**Table:**

Zn1—O1	2.003 (3)	C6—H6A	0.9600
Zn1—N4	2.053 (3)	C6—H6B	0.9600
Zn1—N2	2.063 (4)	C6—H6C	0.9600
Zn1—N3	2.146 (3)	C7—C8	1.502 (6)
Zn1—N1	2.163 (3)	C7—H7A	0.9700
Zn2—O2	1.997 (3)	C7—H7B	0.9700
Zn2—Br3	2.3772 (8)	C8—H8A	0.9700
Zn2—Br2	2.4204 (7)	C8—H8B	0.9700
Zn2—Br1	2.4236 (7)	C9—C13	1.524 (6)
N1—C16	1.474 (5)	C9—C14	1.543 (6)
N1—C1	1.497 (5)	C9—C10	1.548 (6)
N1—H1	0.9100	C10—C11	1.508 (6)
N2—C3	1.264 (6)	C10—H10A	0.9700
N2—C7	1.479 (5)	C10—H10B	0.9700
N3—C8	1.482 (5)	C11—C12	1.508 (6)
N3—C9	1.501 (5)	C12—H12A	0.9600
N3—H3	0.9100	C12—H12B	0.9600
N4—C11	1.266 (5)	C12—H12C	0.9600
N4—C15	1.476 (5)	C13—H13A	0.9600
O1—C17	1.241 (5)	C13—H13B	0.9600
O2—C17	1.272 (5)	C13—H13C	0.9600
C1—C5	1.537 (6)	C14—H14A	0.9600
C1—C6	1.538 (6)	C14—H14B	0.9600
C1—C2	1.539 (6)	C14—H14C	0.9600
C2—C3	1.512 (7)	C15—C16	1.507 (6)
C2—H2A	0.9700	C15—H15A	0.9700
C2—H2B	0.9700	C15—H15B	0.9700
C3—C4	1.501 (7)	C16—H16A	0.9700
C4—H4A	0.9600	C16—H16B	0.9700
C4—H4B	0.9600	C17—C18	1.504 (5)
C4—H4C	0.9600	C18—H18A	0.9600
C5—H5A	0.9600	C18—H18B	0.9600
C5—H5B	0.9600	C18—H18C	0.9600
C5—H5C	0.9600		
			
O1—Zn1—N4	109.13 (13)	H6B—C6—H6C	109.5
O1—Zn1—N2	123.65 (13)	N2—C7—C8	109.9 (4)
N4—Zn1—N2	126.98 (14)	N2—C7—H7A	109.7
O1—Zn1—N3	98.04 (12)	C8—C7—H7A	109.7
N4—Zn1—N3	94.26 (14)	N2—C7—H7B	109.7
N2—Zn1—N3	83.84 (14)	C8—C7—H7B	109.7
O1—Zn1—N1	88.39 (12)	H7A—C7—H7B	108.2
N4—Zn1—N1	83.31 (14)	N3—C8—C7	110.1 (4)
N2—Zn1—N1	92.85 (14)	N3—C8—H8A	109.6
N3—Zn1—N1	173.56 (13)	C7—C8—H8A	109.6
O2—Zn2—Br3	122.76 (10)	N3—C8—H8B	109.6
O2—Zn2—Br2	98.32 (8)	C7—C8—H8B	109.6
Br3—Zn2—Br2	108.54 (3)	H8A—C8—H8B	108.2
O2—Zn2—Br1	104.25 (9)	N3—C9—C13	107.2 (4)
Br3—Zn2—Br1	108.71 (3)	N3—C9—C14	111.6 (4)
Br2—Zn2—Br1	114.23 (3)	C13—C9—C14	109.1 (4)
C16—N1—C1	116.5 (4)	N3—C9—C10	110.5 (4)
C16—N1—Zn1	104.9 (3)	C13—C9—C10	111.3 (4)
C1—N1—Zn1	116.4 (3)	C14—C9—C10	107.2 (4)
C16—N1—H1	106.1	C11—C10—C9	119.4 (4)
C1—N1—H1	106.1	C11—C10—H10A	107.5
Zn1—N1—H1	106.1	C9—C10—H10A	107.5
C3—N2—C7	121.6 (4)	C11—C10—H10B	107.5
C3—N2—Zn1	129.4 (4)	C9—C10—H10B	107.5
C7—N2—Zn1	109.1 (3)	H10A—C10—H10B	107.0
C8—N3—C9	116.2 (4)	N4—C11—C10	121.7 (4)
C8—N3—Zn1	104.7 (2)	N4—C11—C12	123.9 (4)
C9—N3—Zn1	115.5 (2)	C10—C11—C12	114.3 (4)
C8—N3—H3	106.6	C11—C12—H12A	109.5
C9—N3—H3	106.6	C11—C12—H12B	109.5
Zn1—N3—H3	106.6	H12A—C12—H12B	109.5
C11—N4—C15	123.0 (4)	C11—C12—H12C	109.5
C11—N4—Zn1	127.9 (3)	H12A—C12—H12C	109.5
C15—N4—Zn1	108.9 (3)	H12B—C12—H12C	109.5
C17—O1—Zn1	143.7 (3)	C9—C13—H13A	109.5
C17—O2—Zn2	118.5 (3)	C9—C13—H13B	109.5
N1—C1—C5	106.7 (3)	H13A—C13—H13B	109.5
N1—C1—C6	111.3 (4)	C9—C13—H13C	109.5
C5—C1—C6	107.8 (4)	H13A—C13—H13C	109.5
N1—C1—C2	110.9 (4)	H13B—C13—H13C	109.5
C5—C1—C2	110.2 (4)	C9—C14—H14A	109.5
C6—C1—C2	109.8 (4)	C9—C14—H14B	109.5
C3—C2—C1	118.8 (4)	H14A—C14—H14B	109.5
C3—C2—H2A	107.6	C9—C14—H14C	109.5
C1—C2—H2A	107.6	H14A—C14—H14C	109.5
C3—C2—H2B	107.6	H14B—C14—H14C	109.5
C1—C2—H2B	107.6	N4—C15—C16	108.9 (4)
H2A—C2—H2B	107.0	N4—C15—H15A	109.9
N2—C3—C4	123.5 (5)	C16—C15—H15A	109.9
N2—C3—C2	120.8 (5)	N4—C15—H15B	109.9
C4—C3—C2	115.7 (4)	C16—C15—H15B	109.9
C3—C4—H4A	109.5	H15A—C15—H15B	108.3
C3—C4—H4B	109.5	N1—C16—C15	109.5 (4)
H4A—C4—H4B	109.5	N1—C16—H16A	109.8
C3—C4—H4C	109.5	C15—C16—H16A	109.8
H4A—C4—H4C	109.5	N1—C16—H16B	109.8
H4B—C4—H4C	109.5	C15—C16—H16B	109.8
C1—C5—H5A	109.5	H16A—C16—H16B	108.2
C1—C5—H5B	109.5	O1—C17—O2	121.7 (4)
H5A—C5—H5B	109.5	O1—C17—C18	121.9 (4)
C1—C5—H5C	109.5	O2—C17—C18	116.4 (4)
H5A—C5—H5C	109.5	C17—C18—H18A	109.5
H5B—C5—H5C	109.5	C17—C18—H18B	109.5
C1—C6—H6A	109.5	H18A—C18—H18B	109.5
C1—C6—H6B	109.5	C17—C18—H18C	109.5
H6A—C6—H6B	109.5	H18A—C18—H18C	109.5
C1—C6—H6C	109.5	H18B—C18—H18C	109.5
H6A—C6—H6C	109.5		

### Hydrogen-bond geometry (Å, °)

**Table:**

*D*—H···*A*	*D*—H	H···*A*	*D*···*A*	*D*—H···*A*
N3—H3···Br2^i^	0.91	2.80	3.549 (3)	140
N1—H1···Br1	0.91	2.74	3.641 (3)	171

Symmetry codes: (i) −*x*+1, −*y*+1, −*z*.
